# UK science press officers, professional vision and the generation of expectations

**DOI:** 10.1177/0963662515597188

**Published:** 2015-08-11

**Authors:** Gabrielle Samuel, Clare Williams, John Gardner

**Affiliations:** Brunel University London, UK; University of York, UK

**Keywords:** hype, media, press officers, professional vision, sociology of expectations

## Abstract

Science press officers can play an integral role in helping promote expectations and hype about biomedical research. Using this as a starting point, this article draws on interviews with 10 UK-based science press officers, which explored how they view their role as science reporters and as generators of expectations. Using Goodwin’s notion of ‘professional vision’, we argue that science press officers have a specific professional vision that shapes how they produce biomedical press releases, engage in promotion of biomedical research and make sense of hype. We discuss how these insights can contribute to the sociology of expectations, as well as inform responsible science communication.

## 1. Introduction

Innovations in biotechnology and medicine are associated with narratives of breakthrough and discovery. As the ‘sociology of expectations’ has made clear, hype and future-orientated abstractions within such rhetoric are not simply a by-product of innovation. They are, rather, performative. By envisaging futures in the present, they prompt the alliance-building that constitutes the innovation process itself (Borup et al., 2006; Brown, 2003; Michael, 2000). In effect, such ‘breakthrough’ narratives are employed as strategic resources to enrol allies and secure resources. Exploring the generation of positive expectations surrounding biomedical innovations can therefore provide important insights into the dynamics of socio-technical change (Borup et al., 2006). Less-promissory visions of the future (‘low expectations’) also abound, and these too can provide momentum to biomedical innovation (Gardner et al., 2015; Tutton, 2011).

The representation of biomedical innovations in news media provides an excellent case for exploring the generation of expectations. Social science research into the news media’s portrayal of innovative technologies reveals how representations tend to contain ‘breakthrough’ language, proclaim imminent medical benefits and promote hype and expectations (Nelkin, 1996; Racine et al., 2010; Seale, 2003). A closer analysis of news media’s role in generating expectations exposes a political climate in which research and innovation are essential drivers of economic growth and international competitiveness. Funding bodies, research policies and the private sectors place considerable pressure on scientists and research institutions to pitch their accomplishments in optimistic terms so as to secure capital and prestige (e.g. Caulfield, 2005). Consequently, high expectations abound, which are then picked up and disseminated by the media.

Products of this political climate are science press officers (SPOs), whom research institutions employ to manage communication of their accomplishments to the public. SPOs and their press releases have become the focus of social scientists’ attention and there is a fast-growing body of research that links sensationalist news reporting of biomedical technologies to press releases (e.g. see Sumner et al., 2014). Much of this work argues that SPOs and their press releases play a key role in generating expectations and in perpetuating ‘hype’, that is, the promotion of science by extravagant claims and/or the exaggeration of its benefits (Henderson and Kitzinger, 2007; Nerlich et al, 2002; Williams and Gajevic, 2013). Indeed, the press release is the production of research communities seeking to raise the profile of their work, as a means of persuading potential patrons of the benefits of investment or sceptical publics of future benefits (Brown, 2003). Even in an increasingly online world, SPOs and press releases still have a pivotal role in the generation of expectations: the majority of information circulated on online platforms still comes from traditional media, and it is these stories that tend to set the narrative agenda for most other media outlets (Pew Research Center’s Project for Excellence in Journalism, 2010).

To explore the role of SPOs and press releases in the generation of expectations and hype, in this article, we draw on a small set of interviews with 10 UK-based SPOs, collected as part of a larger study on the production and reception of media and press releases in the area of innovative neuroscience, some aspects of which have been reported in Samuel and Kitzinger (2013). Using Goodwin’s (1994) concept, we argue that these SPOs have a specific professional vision that shapes how they engage in the promotion of biomedical research and how they make sense of hype. Towards the end of this article, we briefly draw upon the findings of interviews conducted with members of the public to illustrate that what exactly constitutes ‘hype’ is the subject of contention and is the result of different, or contested, professional visions. The significance of using Goodwin’s professional vision is that it describes different visions of hype, and it also teases out how such perspectives develop through professional practices.

In what follows, we summarise research which has explored the role of SPOs and press releases in generating hype and introduce Goodwin’s notion of professional vision. We present our methods and discuss our findings in light of Goodwin’s concept. We then demonstrate how these findings contribute to the sociology of expectations and conclude by reflecting on the implications for those involved in promoting and representing biomedical research to the UK public.

## 2. Science press officers and their press releases

Changes in the global economic and political climate of science research and science communication over the past 30 years have led to an expansion of science press releases and SPOs. In the United Kingdom, these changes have been attributed to a number of events, including the Royal Society’s 1985 report, *The Public Understanding of Science* and the House of Lords Select Committee on Science and Technology’s 2000 publication, *Science and Society*. Both promoted the importance of communicating science to the public, resulting in a much expanded role for science communicators, and a media which is increasingly interested and orientated towards science (‘mediatisation’; ‘medialisation’ (Rödder, 2009)).

Alongside these events, science institutions are being held accountable to disseminate findings to the public, who may no longer recognise and accept the professional elites’ privilege of unaccountability (Weingart, 2012). They have become more ‘commercial’ due to affiliations with industry, leading to an increased need to market oneself (Rose, 2003); they face increasing competition for limited resources; and they have been steered (e.g. via the Research Excellence Framework 2014) to demonstrate societal impact of their research. Because of these factors, growing media attention to science has been matched by ‘an increasing *orientation of science towards the media*’ (Rödder, 2009: 453) as scientists and institutions market themselves to the public and stakeholders to garner support for their research. This orientation has taken the form of a rapid professionalisation and expansion of SPOs (Peters et al., 2008; Schäfer, 2011). SPOs are now considered an integral part of the science research dissemination process. Stempra (2009) – The Science Technology Engineering and Medicine Public Relations Association – describe SPOs’ position as follows:

As press officers and science communicators, we often act as the brokers in the knowledge exchange between scientists and journalists. We help journalists make sense of complex science and help scientists make the complex science make sense. (p. 3)

Stempra’s (2009) ‘Guide To Being a Press Officer’ describes the aim of SPOs as to ‘*generat[e] interest in a story*’, while at the same time to ‘*communicate science responsibly*’ and ‘*not over-sell*’ (p. 3). This reflects SPOs’ recent history of expansion, driven by increasing science communication projects as well as research institutions’ needs to publicise.

Press releases are a key means whereby SPOs disseminate science research to journalists, and frequently, they are the main source of access media journalists have to biomedical research being conducted in science institutions. In spite of the growing trend in using online modes for science communication, studies within the United Kingdom (Castell et al., 2014), as well as in Europe (TNS Opinion and Social, 2013) and the United States (Schoen, 2008), suggest that most individuals learn about science from traditional media and that the content of online media traditionally draws on traditional sources (Pew Research Center’s Project for Excellence in Journalism, 2010). While online, more interactive modes of communication hold future promise, asymmetrical models of science communication using the press release are still the preferred method used by SPOs (Borchelt and Nielsen, 2008).

Research has shown that expectations attached to innovative biomedical technologies in the news media can be directly attributed to the press releases disseminated to publicise the research (Brechman et al., 2009; Racine et al., 2006; Sumner et al., 2014; Woloshin and Schwartz, 2002). SPOs have also been shown to have an integral role in the generation of expectations – through studies that have analysed the perspectives of science communicators in general (Bubela et al, 2009) and/or via an examination of large-scale, costly ‘big science’ stories (Henderson and Kitzinger, 2007; Nerlich et al., 2002; Williams and Gajevic, 2013). Thus, the role of SPOs as being able to ‘*communicate science responsibly*’ has been questioned by scholars, who argue that such communication is inconsistent with the marketing nature of the position (Borchelt and Nielsen, 2008).

## 3. Goodwin’s ‘professional vision’

Goodwin’s seminal article on professional vision employed linguistic anthropology and conversation analysis to examine the way in which professions make sense and articulate their understanding of events within the world. Goodwin argues that any event in the world is composed of a near-infinite number of phenomena. Actors construct their understandings of such events by foregrounding some phenomena as ‘relevant’ and by drawing these phenomena into an account of the event. The particular phenomena that are deemed relevant, and the way in which these are assembled into an account, will depend in part on the actor’s professionally acquired perspective. It is possible for actors from different professions to have divergent understandings and explanations of the same event. Goodwin illustrates this with the example of conflicting interpretations of the events within the Rodney King Videotape. This videotape became a politically charged theatre for contested vision during the 1992 US trial of four White policemen charged with beating Mr King, an African–American motorist. Opposing sides of the case used the same videotape to demonstrate very different events. One argued that the videotape was evidence of a savage beating of a docile suspect; the other (adopting the ‘vision’ of the police officers) used the videotape as evidence of a careful police response to the threatening actions of a dangerous man.

Goodwin argued that that the way individuals are trained in their profession affects how those individuals perceive the world: they interpret the world in an occupationally specific manner which relates to the ‘*body of practices through which the objects of knowledge which animate the discourse of a profession are constructed and shaped*’ (Goodwin, 1994: 605). Goodwin (1994) states,

Different professions … have the power to legitimately see, constitute and articulate alternative kinds of events. Professional vision is perspectival, lodged within specific social entities …[…] … such vision … is something accomplished through the competent deployment in a relevant setting of a complex of situated practices. (p. 626)

Those situated practices, argued Goodwin, can be divided into three components: coding schemes (processes of classification), highlighting (processes of selecting material) and the production and articulation of material representations (the ability to view and report material through the ‘eyes’ of a specific profession).

While the SPO role does not strictly fulfil the criteria of a profession in sociological terms (e.g. there is no specific course/exam which needs to be completed and no official membership), they do loosely represent a community characterised by, for instance, a particular expertise, language and tools (Fournier, 2000). SPOs have a unique job, characterised by very specific tensions. Below, we use the findings of our study to demonstrate how Goodwin’s ‘professional vision’ could be a useful concept for understanding how SPOs construct their views and beliefs around science reporting. We analyse our data in relation to Goodwin’s three concepts of situated practice.

## 4. Materials and methods

As part of a broader media analysis project, 10 SPOs (eight females, two males) were recruited from a number of biomedical institutions: industry (*n* = 1), science journals (*n* = 1), science/medicine funding bodies (*n* = 2), charity organisations (*n* = 2), university science departments (*n* = 2), and media centres (*n* = 2). All interviewees were experienced: seven had been in the profession for between 4–9 years, while three had over 20 years experience. Most interviewees had worked in several different institutions as SPOs and had accumulated a variety of experiences. For example, one interviewee drew on experience from the UK National Health Service (NHS), a medical charity organisation, and a research council; another interviewee brought experiences from a research council, a charity association and a business. Ethics approval was obtained from Brunel University London. Informed consent procedures were conducted with all participants.

Interviews were conducted either face-to-face or via the telephone. Novick’s (2008) review of the literature concluded that there is little evidence that data loss or distortion occurs, or that interpretation or quality of findings is compromised, when interview data are collected by telephone. Interviews were semi-structured and were conducted by the first author in 2012. The purpose was to explore participants’ perspectives about the choices they make when choosing which scientific stories to highlight to journalists and why; their perspectives about how they report science; and their views about the role of press releases as a tool for science communication. Obviously such interview data do not provide a lens into the ‘truth’, but rather, to some degree, represent a constructed perspective of reality. This is a key limitation of any interview methodology.

First, interviewees were questioned about their employment background and their current job role. They were asked to describe how they chose stories for press release; how they transform the story into a press release; and how they disseminate the story to journalists. Second, interviewees were shown and asked to comment on a press release and a news article, – both of which reported a study of an innovative neurotechnological application, which used functional magnetic resonance imaging (fMRI) in an attempt to assess brain activity in severely brain-injured patients (Monti et al., 2010). The news article, which was published in the UK Mirror newspaper, was selected because of its sensationalist headline, large ‘visual’ section with pictures of brain scans, and main article which reported the study using mobilizations of hope, excitement and expectation. As part of a larger qualitative study which has been reported in Samuel and Kitzinger (2013), these same news items were presented to relatives of individuals with a severe brain injury in a series of interviews. We draw on some of the findings from these interviews to act as a comparison with the SPO interviews. All interviews were recorded and transcribed. Transcriptions were thematically analysed using NVivo.

## 5. Results

During our interviews, SPOs spoke about the aspects of their role specifically revolving around the production of press releases. Other features of their role, such as those related to online forms of communication, were touched upon, and various views were expressed with relation to this. However, it is the role of SPOs in relation to the press release which is the subject of this article, and what we will be focussing on below.

Throughout our interviews with SPOs, participants often adopted different positions when describing their views regarding the role of an SPO. These positions could be understood, at least in part, in terms of the different agendas of SPOs at their institutions. For example, one participant viewed her role as a promoter, commenting: ‘*the overall overarching … reason I do my job, or kind of why my job is here, is to promote the research at the university …’* (PO1 charity association), whereas another interviewee was more interested in accurately communicating science: ‘*that’s why we’re (SPOs) here – to make sure that ultimately the science gets covered in sort of, as accurate and responsible way as possible*’ (PO 10). In spite of the fact that interviewees had different views on how much their role included promotion or communication (which we discuss in more detail below), two points quickly became evident. First, although some participants substantially leaned one way or another, all participants spoke throughout their interviews about the importance of both the selling aspect, and the science communication aspect, of their role. Second, while participants were aware of the fact that science research can be sensationalised in newspaper reporting, participants did not see themselves as the perpetrators of this ‘hype’. Rather, all interviewees viewed it as their *obligation* to produce accurate, ‘honest’ press releases so as to avoid any unnecessary exaggeration of science, which could potentially lead to false hope for patients.

In the sections ‘Production and articulation of material representations’ and ‘Coding schemes and highlighting’ and ‘Press release as an object of contested vision’, we explore Goodwin’s notion of professional vision through his three ‘situated practices’ and relate it to these findings to help explain how SPOs construct their beliefs about their role in science reporting. While professional vision is constituted by all three situated practices, it is the production and articulation of material representations that Goodwin discussed in most detail, and it also proved the most relevant in helping us understand how SPOs view their role. Therefore, it is this situated practice which we consider first, and which we focus on in most depth.

## 6. Production and articulation of material representations

Goodwin notes that graphic representations are a key factor in understanding professional practice. He uses the graphic representation of an archaeologist’s map and the tools archaeologists associate with map drawing to explain this situated practice. Goodwin (1994) argues ‘*the ability to see in the very complex … landscape … those few events that count as points to be transferred to the map, are central to what it means to see the world as an archaeologist*’. Through their training, all archaeologists share a common perception of ‘*what and where to measure*’ (p. 615) that is specific to their profession, as well as the tools and interactions embedded within it.

While the role of an SPO does not necessarily require the production of graphic illustrations, the science press release can be viewed as analogous. Similar to how an archaeologist-shaped perspective of the landscape results in her/his ability to create an ‘archaeologist map’, an SPO-shaped view of her/his landscape (science research) allows SPOs to produce a press release (their archaeologists’ map) with specific profession-specific features. A science press release is a balancing act between ‘selling’ (to journalists) the newsworthiness of a piece of research in terms of its benefits, while communicating the research responsibly. This tension is indicative of any ‘map’ of a press release, as previously noted in Stempra’s (2009) guide to being a press officer (‘*to walk the fine line between generating interest in a story and over-selling it*’; p. 3). Many participants explicitly highlighted these tensions in their interviews. Participant PO 3 (university) remarked,

We try and do a very honest job… we try not to overstate something but obviously there is always a balance between that, and knowing that in order for the media to be interested in something you need to know what the most compelling aspects of that research are.

You’re trying to portray an accurate overview of the thing that you’re trying to sell, you’re trying to not over hype it too much. (PO 9 journal)

For interviewees, managing this tension was seen as an *obligation* to ensure they did not produce a press release that was hyped:

I certainly feel that they [press officers] have an obligation to write press releases that do not hype the research because that just gets everyone into trouble then and creates false expectations. (PO 2 charity organisation)

We play quite a major role in how things are portrayed and making sure that things aren’t over hyped. (PO 9 journal)

Participants had a clear sense of the potential impact hyping press releases could have on individuals’ lives in terms of hopes and expectations, and spoke explicitly of this:

People who are very sick – or families of people who are very sick – are also desperate and will cling on to any hope, and that’s why you’ve got to manage the situation, and if you do publicise it be prepared for that follow up – you can’t just publicise it and then run away from it. (PO 2 charity organisation)

Goodwin’s production and articulation of material representations (i.e. the practices related to how a ‘map’ of the landscape is produced), is key to understanding how SPOs balance this tension when writing press releases to ensure they represent research responsibly. In Goodwin’s terms, we need to explore what information SPOs look for (in their landscape of science research) to incorporate into their press release (their ‘map’) to ensure their final document represents a press release indicative of their profession (i.e. the content balances tensions between ‘responsible science communication’ and ‘selling’, and does not over-hype research).

For participants, responsible communicating of science research was linked to three factors. First, the accuracy of the facts contained in the press release. According to the situated practices of an SPO, as long as the facts in a press release are accurate then the press release itself is also considered accurate. These facts, however, may be written in a language and style that adopts a selling slant. In fact, ‘*being sensationalist and accurate shouldn’t be mutually exclusive*’ (PO 4 research council):

He [press officer] may say, ‘it’s amazing, it’s astonishing, it’s good they’ve done these things’, as long as he’s got the actual study accurate and what they have and haven’t found. (PO 5 media centre)

Second, the incorporation of the research caveats and limitations in the press release. The placement of caveats at the end of the article was reasonable because, as interviewee PO 10 (media centre) remarked,

When I read … I always read the last bit first because that’s where all the caveats and stuff generally are … I generally don’t read the headlines – I mean obviously you get drawn to it, but I don’t pay attention to them, so … I guess my perspective is very different.

Interviewee PO 5 (media centre) supported this: ‘*I would expect something a bit caveated in the l*ast* paragraph*’.

Third, the use of quotations marks. For interviewees, quotation marks symbolised ‘non-fact’ comments, and for them there was a clear separation between these ‘non-fact quotes’, as opposed to ‘non-quoted facts’ in the rest of the article, for example,

If an academic wants to [elaborate on] some aspects of the research, that’s what a quote is for, so it is very clear that this is nothing … this is something that they feel, but it’s not stated as such in the paper. (PO 6 university)

This interviewee did not view the use of these quotes as having a substantial impact on the ‘balance’ of the reporting (*‘we do use external quotes … typically from funding bodies … I mean, it’s just a quote …’*) but rather, as just highlighting an expert’s opinion.

This way in which our interviewees viewed the responsible communication of science can be compared with the views of those outside of the profession (e.g. the public). First, while our participants viewed quotation marks as a place for non-facts and elaboration, it is questionable whether the public distinguishes between what is written in quotes and what is not. Indeed, Fiona Fox, director of the UK Science Media Centre (a national science press office encouraging accurate science news coverage) picked up on this issue in one of her recommendations to the Leveson Inquiry in which she stated, ‘*quotation marks should not be used to dress up overstatement*’ (Fox, 2012). Second, while our participants did not consider that the language style of the article is likely to have an influence on the way the public reads and understands it, this has been disputed by a number of science communication scholars (Bubela, 2009). It has also been noted by Samuel and Kitzinger (2013) in their comparison of news reporting of fMRI research for severely brain-injured individuals with the views of the relatives of severely brain-injured individuals. This study showed that how a news article was written did, in some instances, influence how the relatives viewed the technology. Finally, while for the SPO interviewees the presence, but not necessarily the location, of caveats is important, journalists have repeatedly noted that it is unlikely that the public read a whole news article, and hence the caveats at the end may go unread (e.g. Wastler A, 2013).

Overall, then, we can view the science press release as a ‘map’, the key feature of which is to balance responsible science research reporting with the marketing of this research. This is achieved in a number of ways including the accuracy of facts, the inclusion of caveats, the use of quotes and the style of writing. These situated practices, or what could be called ‘tricks of the trade’ entail a specific perspective on press releases. This was most neatly summed up in one interviewee’s comments about science reporting:

When I look at that [newspaper article] I guess my perspective is very different – in the same way a scientist would pick up a scientific paper and go, ‘well, where are the caveats? Where are the limitations?’, and would break down the paper, I would do that with the article … […] … I don’t know if I’ve been in the job too long because I think had I been still in the middle of my neuroscience PhD I would … go, ‘Oh my God, its hideous’ But I don’t look at it like that now. (PO 10 media centre)

## 7. Coding schemes and highlighting

Alongside the practice of producing and articulating material representations, Goodwin’s situated practices of coding and highlighting are also key to understanding our SPOs interviewees’ professional practice. Goodwin’s (1994) ‘coding schemes’ explain how professions interpret and organise information (in an SPO’s case, all science research) as they ‘*transform the world into the categories and events that are relevant to the work of the profession*’ (p. 608). Using the example of archaeologists, Goodwin (1994) argues how coding schemes are used:

To organize the perception of nature … within the discourse of the profession … [and that they] have far reaching impact, [for example, by dictating] the parameters of that work [which] have been established by the system that is organizing their perception. (p. 609)

Goodwin’s notion of ‘highlighting’ refers to the processes by which workers identify some aspects of information as important and relevant to their profession. Goodwin (1994) states that when a profession is faced with many different documents and texts, workers

Highlight their documents [to] tailor the document so that those parts of it which contain information relevant to their own work are made salient … through these practices structures of relevance in the material environment can be made prominent, and thus become ways of shaping. (p. 610)

Goodwin argues that highlighting and categorising therefore have ‘*powerful persuasive consequences*’ (p. 610) since they guide the perception of others in the profession with respect to how to view, highlight and categorise the landscape, while further reifying previously established categories.

Our interviews provided insight into how our participants highlight and code research and what aspects of a particular piece of science research they view as relevant. First, in terms of coding science research into categories, interviewees primarily did this by its degree of newsworthiness. Given the role of SPOs, unsurprisingly, the rationale for this categorisation of research was explained in terms of the needs of journalists: ‘*I write releases for journalists*’ (PO 1 charity association); *‘you have to remember the audience is the journalist*’ (PO 10 media centre). In fact, the coding scheme participants drew upon to make decisions about which science research to press release reflected those of journalists: *‘You look for … typical journalist components about what makes a story*’ (PO 4 research council). Interviewees spoke about these ‘components’:

There’s a number of criteria that a journalist would apply … for one thing, it has to be new … they have to be able to say, ‘a study published today’. (PO 3 university)

Participants spoke about the importance of a story containing what journalists call the ‘so what’ factor:

People talk a lot about the ‘so what’ question. It’s essentially, what does this mean to the general public … if you can’t answer that there’s no point in publicising it. (PO 5 media centre)

This reliance on journalists was most clearly illustrated when interviewees discussed how journalists’ views were the driving force in the production of specific guidance documents for their industry. Interviewee PO 7 (research council) commented,

They’ve got a guide to working for press officers and I edited that, and in the course of it I spoke to quite a few journalists, asked them what they wanted in a press release … we basically want to make it as easy as possible for the journalists to write up a story.

Second, interviewees revealed how they highlight relevant information about a specific research study which had been chosen for press release. This was most evident when interviewees discussed the meetings they had with researchers as they drafted releases. Participants spoke about requiring specific pieces of information from researchers, which could then be ‘highlighted’ in a press release. For example, ‘*why their research is so important to that particular field, future direction of their research and their goals, the number of people who are suffering from the disorder*’ (PO 2 charity organisation). This is illustrated as PO2 explains further how, even if basic science is being reported, what needs to be highlighted in the press release are the key aspects of that disease:

If you were writing about allergy and asthma, it may be basic science but if you can say, ‘well, x number of people suffer from asthma’, again, it’s putting it in context – the potential clinical benefits of the research … (PO 2 charity organisation)

Similar to above, participants’ professional vision of ‘highlighting’ was directly in line with that of journalists. When choosing aspects of research to highlight in press releases, this was contextualised in terms of what journalists needed in their own work: ‘*we tend to write it so [the journalists have] got all the information in front of them*’ (PO 7 research council).

Participants also explained how the information they had obtained from researchers needed to be structured into a science press release in a specific manner – certain pieces of information from research were required to be highlighted at the top of the release, while other aspects of the research could feature further down. Interviewees frequently used journalists’ narratives to also explain this: they spoke about the journalists’ standard news pyramid (a metaphor used by journalists to illustrate how information should be prioritised and structured in a text) and about a ‘grabbing’ headline:

There’s a fairly easy schematic to look at if you’re looking at any press release. You go for the inverted triangle…we work on the same principle [as journalists], so we expect to get anybody’s attention in our first paragraph – always answering who, what, where, why and when … – and then go into the methodology … afterwards. (PO 4 research council)

Overall, as Goodwin notes, both coding schemes and highlighting have wide-reaching consequences. Thus, press releases will be written and shaped by which aspects of science research our SPO participants deem ‘valuable’ enough to highlight versus which aspects they pay less interest to – for example, the questioning and close analysis of the methods, assumptions, concepts and ideas used in the research. The fact that our participants’ coding schemes are based upon, and aimed at, journalists has implications on how they view science reporting and the nature of ‘hype’ as a whole.

## 8. The press release as an object of contested vision

Goodwin (1994) argued that, because the Rodney King videotape was used by opposing sides to argue very different cases, it ‘*became a politically charged theatre for contested vision*’ (p. 606). The videotape was viewed as an object of knowledge by both parties – but because of their differing professional visions, they had contested understandings of what the videotape revealed.

We have shown that our participants have a specific professional vision that informs how they construct press releases and how they define ‘hype’. Because of this, their understanding of ‘responsible’ communication and ‘hype’ will differ from that of other professions and individuals. Indeed, we propose that there is a *contested vision* between how our SPO participants view a science press release (i.e. whether it is hyped) and how others view that same press release. In this section, we will illustrate this by drawing on focussed discussions we had with interviewees about a press release and a news article reporting on the use of an innovative neurotechnology on a patient who had a severe brain injury. Comparing these discussions with a scholarly analysis of the press release and news article, along with similar discussions with potential users of the technology about the news article – both of which have been reported previously in the literature (Samuel and Kitzinger, 2013) – provides an illustration of the contested visions and differing understandings of hype.

**Figure 1.**
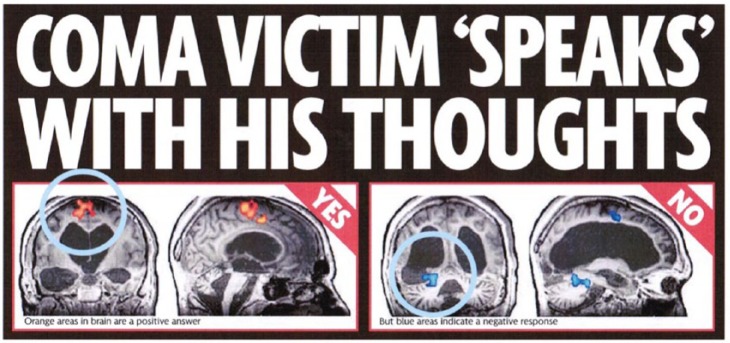
Headline and visual section of the Mirror article reporting the fMRI research.

The press release and news article reported the use of fMRI in an attempt to diagnose and communicate with severely brain-injured patients. The study received wide media coverage, and scholars noted that the press release and news article presented optimistic portrayals using the language of ‘breakthrough’, with a focus on the benefits (Samuel and Kitzinger, 2013) (see Figure 1). Furthermore, during interviews conducted with individuals who have a relative with a severe brain injury, some interviewees also viewed the news article as overly optimistic: ‘*cause this [the fMRI news article] makes you think that you’re going to speak to them [severely brain-injured relative] through this magic machine*’ (interviewee) (Samuel and Kitzinger, 2013). In contrast, our SPO interviewees generally viewed the fMRI newspaper item as well written: ‘*I think this is quite good*’ (PO 10 media centre); and ‘*it does have caveats in there … saying it is early stages … I would have [been] happy with that one*’ (PO 4 research council). Interviewee PO 5 (media centre) was very positive about the article, commenting that it was a ‘*big deal*’. In fact, PO 5 commented,

If this doesn’t sell they’ve found anything more amazing than what’s actually in the original study that’s good reporting, he may say it’s amazing, it’s astonishing, it’s good they’ve done these things, as long as he’s got the actual study accurate and what they have and haven’t found.

Participants viewed the newspaper report as an accurate reflection of the release: ‘*it seems a pretty accurate reflection really*’ (PO 2 charity organisation); ‘*overall I think his tone reflects the press release*’ (PO 5 media centre); and ‘*from the press release, it’s fine actually the way they’ve written it*’ (PO 7 research council). Only one interviewee voiced serious concerns about the article content:

It’s a shame that articles don’t put success rates, or how small the sample was, or how many people out of how ever many it actually worked for … If it’s just one case study there’s no way of knowing whether it’s transferable into others and they’re not highlighting that at all, they don’t outline the reasons why perhaps further research is needed or perhaps we should be cautious … they should put things like that in there so people can have a more balanced view about how realistic this is. (PO 9 journal)

These findings allude to a contested vision: while most of our interviewees viewed the fMRI reporting as relatively unproblematic, this was not necessarily the case for scholars and/or potential users of the technology.

## 9. Conclusion

This article suggests that Goodwin’s notion of professional vision is a useful concept for exploring the manifestation of hype in UK biomedical research news reporting. In particular, through this small-scale study with UK SPOs, we have endeavoured to demonstrate how ‘hype’ is the product of a professional vision, which is itself the result of ‘*the competent deployment in a relevant setting of a complex of situated practices*’ (Goodwin, 1994: 626). For our participants, achieving a ‘balanced’, ‘non-hyped’ press release was linked to a number of factors relating to, for example, accuracy and the prevalence of caveats. The language style of the press release, for instance, was not viewed as something which contributed to ‘hype’, but rather allowed press releases to appeal to journalists. Such situated practices allowed them to ‘*walk the fine line between generating interest in a story and over-selling it*’; Stempra, 2009: 3).

Our findings have implications both theoretically and practically. Theoretically, they may contribute to the sociology of expectations in relation to new biomedical technologies. This literature has explored in detail how expectations are generated (Borup et al., 2006; Brown, 2003), as well as the role of SPOs and press releases in the generation of these expectations (Henderson and Kitzinger, 2007; Kitzinger, 2008; Nerlich et al, 2002). In particular, our use of Goodwin’s ‘professional vision’ has enabled us to tease out, at least for the UK perspective, how perspectives towards biomedical science reporting and the generation of expectations become embedded within, and are a product of, particular professional practices.

Thus we argue that Goodwin’s notion of professional vision provides a useful framework to explore how ‘cycles of hype’ actually emerge from science communication practices. The role of SPOs can be characterised by unique situated practices for highlighting, coding and articulating representations. These generate a press release which, according to the SPO professional vision, may be viewed as a realistic, neutral account of science presented in a fashion to attract readers, but to others, may appear as hyped and misleading. Goodwin’s notion of professional vision is part of a much broader body of work which demonstrates that expertise and practices of seeing are locally organised and tied to the particular practical concerns of actors (Heath and Luff, 2000; Lynch, 1985). This body of work demonstrates that it is only by being embedded in such locally organised ‘practices of seeing’ that documents acquire meaning and can become the source of further action; they do not contain any inherent, independent or ‘truthful’ meaning on their own (Perrotta, 2012). Similarly, a press release is not inherently ‘alluring’, ‘neutral’ or ‘hyped’. It has, rather, interpretive flexibility: a capacity to be understood as being any of these qualities depending upon the professionally and culturally acquired ‘practices of seeing’ of those individuals and groups interacting with it. Indeed, in this regard, hype can be understood as a consequence of the interpretive flexibility of documents, such as press releases, as they circulate between different groups and publics. Bringing these concepts to the body of work on the sociology of expectations makes, we argue, a useful contribution to the literature. Further research will allow us to determine if such a contribution extends further afield, outside the area of biomedical science, and also internationally.

This article is contextualised within biomedical research news reporting in the United Kingdom, and it is here where our findings have implications in terms of policy. While the sociology of expectations refrains from making normative judgments about hype in science news reporting, many other scholars have raised various concerns about the expectations which abound in such reporting, especially in the health and technology arena (e.g. see Bubela et al., 2009; Nerlich et al., 2002; Schäfer, 2011; Sumner et al., 2014). Many are concerned that such reporting misleads the public about important issues and leads to false hopes about specific ‘medical breakthroughs’ (Weimann and Lev, 2006). Indeed, a recent working article on novel neurotechnologies published by the UK’s Nuffield Council on Bioethics, raised concerns about the over-optimistic portrayal of novel neurotechnologies in the news media, and the potentially detrimental consequences of such presentations. It included a series of recommendations:

We recommend that all actors working in professions involved in communicating the findings of research involving novel neurotechnologies have a responsibility to reflect upon how their representation of the current and future applications of novel neurotechnologies might impact on others and to remain circumspect about the promises of these applications (however exciting they may be to them professionally or personally). (Nuffield Council on Bioethics, 2013: 218)

The report lists a number of ways in which this can be achieved, for example, to ‘resist pressure to publish only positive…findings’; ‘to be aware of the broader social, legal, and political implications … ’ and ‘to reflect on the pressure … to add a “pinch of hype” and to consider the successive and cumulative effect of this …’ (Nuffield Council on Bioethics, 2013: 219).

Drawing on our findings, while the intentions of the report are laudable, we suggest that it fails to take into account the contested nature about what ‘hype’ is. Our participants are only too aware of the consequences of ‘hype’ on patients, families and members of the public, and participants spoke explicitly about this during their interviews. And while UK SPOs may only publish positive findings and may – in the view of others – add a ‘pinch of hype’, our findings suggest that UK SPOs do not envisage themselves as the generators of hype. By enacting their situated practices, they see themselves as accurately reporting facts and caveats while making their press releases appealing to journalists. Because of this, asking UK SPOs to reflect further upon their work is unlikely to have much effect. Since we cannot define the hype surrounding novel neurotechnologies singularly or unproblematically, we would therefore argue that to address current concerns about ‘hype’ in such reporting would require much more thought and questioning, and possible re-analysis and re-configuration, of the fundamental structures, professions and agendas involved in disseminating science to the public.

Such policy implications may or may not be specific to a UK perspective. UK science communication is moderated by other conventions not shared in other countries, for example, the UK Science Media Centre. In addition, the United Kingdom formally assessed the societal impact of research on a national scale for the first time in 2014 as part of the Research Excellence Framework (REF), and while this was not mentioned by our participants, it is likely that the exercise increases pressure on many UK institutions to achieve ‘impact’, and therefore, in turn, places additional pressure on UK SPOs. In saying that, we note that press releases are a well-established international science news source viewed by institutions as an effective means of communicating science (Autzen, 2014; Weitkamp, 2014). And while there is still a paucity of empirical research exploring the role of SPOs outside of the United Kingdom, a number of international commentaries about the role of SPOs (e.g. see Pantarotto and Jori, 2007) suggest that our findings may have wider relevance.

Overall, this particular article reports on a small collection of UK-based SPOs. Additional studies into the situated practices and understandings of SPOs and the public are now needed to determine whether Goodwin’s concept of ‘professional vision’ can be applied widely across the UK SPO profession, and to generate a nuanced understanding of the social and political implications of science and science and communication at an international level. Such an understanding will permit more effective policy making.
